# Urinary IL-8 is a marker of early and long-term graft function after renal transplantation

**DOI:** 10.1080/0886022X.2017.1323644

**Published:** 2017-05-11

**Authors:** Ewa Kwiatkowska, Leszek Domański, Joanna Bober, Krzysztof Safranow, Jolanta Szymańska-Pasternak, Aneta Sulecka, Andrzej Pawlik, Kazimierz Ciechanowski, Sebastian Kwiatkowski

**Affiliations:** a Clinical Department of Nephrology, Transplantology and Internal Medicine, Pomeranian Medical University in Szczecin, Szczecin, Poland;; b Department of Medical Chemistry, Pomeranian Medical University, Szczecin, Poland;; c Department of Biochemistry, Pomeranian Medical University, Szczecin, Poland;; d Department of Physiology, Pomeranian Medical University, Szczecin, Poland;; e Department of Obstetrics, Pomeranian Medical University, Szczecin, Poland

**Keywords:** IL-8, MMP-9, kidney, ischaemia–reperfusion, graft

## Abstract

In this study, we examined whether the IL-8 content of urine sampled on day 1 and day 14 after renal transplantation is a marker of early and long-term renal function. Moreover, we assessed whether its concentration is positively correlated with the matrix metalloproteinase-9 (MMP-9) content of urine sampled on day 1 and day 30 and 12 months after renal transplantation. Our analysis covered 87 patients who underwent a kidney transplant. The patients were observed for an average of 30 months (12–60 months). The IL-8 concentration determined on day 1 was significantly negatively correlated with creatinine clearance early after renal transplantation (on days 1, 7, 14 and 30), as well as during long-term observations. IL-8 concentration in urine sampled on day 1 and day 14 was higher in patients demonstrating DGF than in those without DGF. No relationship was found between IL-8 content and cold ischaemia time. MMP-9 activity determined on day 1 and month 3 after renal transplantation was positively correlated with the IL-8 content determined in urine sampled on day 1, Rs = +0.32, *p* < .05 and Rs = +0.31, *p* < .05, respectively. The results of this study suggest that a high IL-8 content in urine sampled on day 1 after renal transplantation is an unfavourable marker of early and long-term (years-long) graft function. A high IL-8 content in urine sampled on day 1 after renal transplantation was positively correlated with the activity of metalloproteinase-9 in urine. This proves that both of these chemokines cooperate in ischaemia–reperfusion injuries in transplanted kidneys.

## Introduction

Renal ischaemia–reperfusion (I-R) injury (IRI) is an unavoidable feature of organ transplantation and may have a negative impact on the graft, its function and survival. This process is still being studied. The scale of renal I-R injury is determined by the exacerbation of the inflammatory process it induces. Recently, it has been shown that the peritubular endothelium is the target and source of inflammation in the I-R mechanism [1–3]. Ischaemia causes damage to endothelial cells, which upregulates various adhesion molecules, such as ICAM-1, P-selectin and E-selectin [4,5]. These favour neutrophil adhesion and migration to the extravascular space. Having found themselves in the extravascular space, the neutrophils release free radicals, proteases and elastases, causing a further increase in vascular permeability, which enables other inflammatory cells, such as macrophages, B cells and T cells, to migrate to the renal parenchyma. This allows for a further spreading of the inflammatory process [6,7]. The factors that activate neutrophils, and also facilitate their and other inflammatory cells’ migration, will determine the scale of the IRI and the early and long-term function of the transplanted kidney. IL-8 is the main neutrophil chemokine that is produced by the damaged endothelium and, after inflammation spreads, by macrophages and renal tubular epithelial cells [8,9]. Matrix metalloproteinase-9 (MMP-9) is involved in indirect granulocyte activation and facilitates their migration. Firstly, it is an activator of IL-8, which is activated by the cleavage of a short amino acid sequence. Secondly, it activates a peptide originating from endothelial cells that activates neutrophils. MMP-9 is a proteolytic enzyme and a member of the gelatinase group. Such enzymes are primarily involved in digesting proteins that are components of the extracellular matrix (ECM) and the basement membrane of glomerular and peritubular vessels. By digesting the basement membrane, they destroy the physiological barrier that makes it impossible for cells to migrate from the vascular lumen to the extravascular space.

Plasma and urine concentrations of MMP-9 and IL-8 have been examined in various kidney diseases. Bauvois et al. have shown decreased plasma MMP-9, concentrations in IgA nephropathy and membranous nephropathy [10]. In contrast, Endo et al. have indicated increased serum MMP-9 concentrations in immunoglobulin A nephropathy and other nephropathies [11]. It has been shown that IL-8 plays a significant role in inflammatory pathways leading to kidney allograft rejection [12–14].

In this study, we examined whether the IL-8 content of urine sampled on day 1 and day 14 after renal transplantation is a marker of early and long-term renal function. Moreover, we assessed whether its concentration is positively correlated with the MMP-9 content of urine sampled on day 1 and day 30, as well as after 12 months, after a renal transplant.

## Materials and methods

### Patients

Our analysis covered 87 patients, who underwent a kidney transplant in the Clinical Department of Surgery and Transplantology, Pomeranian Medical University in Szczecin, between 2006 and 2008. They were subsequently under the care of the Clinical Department of Nephrology, Transplantology and Internal Medicine. The patients were observed for an average of 30 months (12–60 months). Unfortunately, not all of the patients were subjected to long-term observation, as some of them changed transplantation centre. Our observations included attending to the patients after their kidney transplantation procedure and making regular follow-up appointments throughout their entire post-transplant period. After kidney transplantation, all of the patients received triple immunosuppressive therapy with glucocorticosteroids, a calcineurin inhibitor (cyclosporine plasma concentration 100–300 ng/mL, tacrolimus plasma concentration 5–15 ng/ml) and mycophenolate mofetil (2 g/24 h).

### Methods

After kidney transplantation, urine was sampled on day 1 and day 14 to assess IL-8 content and on day 1, day 30 and after 12 months to assess MMP-9 content. In patients who could not urinate due to delayed graft function, the first sample was collected when the amount of urine exceeded 500 mL per day. Urine samples were centrifuged at 4000 rpm for 10 min, and sediment-free urine was stored at −80 °C prior to analysis. IL-8 content was determined with an enzyme-linked immunosorbent assay (ELISA) using R&D Systems USA kits (D8000C), with the application of specific polyclonal antibodies. The assay sensitivity was 1.5 pg/mL. For all readings, an ELx808 microplate reader by BIO-TEK Instruments, Inc, Winooski, VT, was used. The MMP-9 content was determined with an enzyme-linked immunosorbent assay (ELISA) using R&D Systems USA kits (DMP900), with the application of specific polyclonal antibodies. The assay sensitivity was 0.156 ng/mL for MMP-9. For all readings, an ELx808 microplate reader by BIO-TEK Instruments, Inc, Winooski, VT, was used.

For each patient, we determined the levels of creatinine and urea in their blood serum. In addition, a general urinalysis was carried out and calcineurin inhibitor content was identified when the urine was sampled during each follow-up appointment. Moreover, information regarding the donor’s details, cold ischaemia time, HLA and PRA (panel reactive antibody) disparity, and the recipient’s details, such as the cause of renal failure, the duration of renal replacement therapy, the patient’s sex, age, weight, the occurrence of delayed graft function (DGF) and the need for haemodialysis (HD) within 7 days of kidney transplantation, were analysed. Furthermore, some of the monitored patients received protocol biopsies at month 3 and month 12. For patients with delayed graft function, biopsies were performed during the first 14 days (Table 1).

**Table 1.** Clinical characteristics of the renal transplant recipients studied.

Characteristic	*N*	Median	Mean ± SD	Range
Time of observation [months]	87	36	31.8 ± 21.54	0.5–60
Age [years]	87	49	45.56 ± 14.68	18–80
Dialysis before Tx [months]	70	20	23.8 ± 18.16	16–75
Residual diuresis [ml]	75	300	664.84 ± 857	300–3000
Weight [kg]	71	72	70.9 ± 13.01	72–98.5
CIT [hours]	72	20.5	21.45 ± 9.01	0–42
Mismatch A	70	1	1.16 ± 0.7	0–2
Mismatch B	70	1	1.34 ± 0.68	0–2
Mismatch DR	70	1	0.71 ± 0.68	0–2
HLA points	66	12	12.34 ± 4.2	2–19
PRA [%]	61	0	3.44 ± 7.1	0–40

SD: standard deviation; Tx: transplantation; CIT: cold ischaemia time; PRA: panel reactive antibody.

### Statistical analysis

We used Statistica 9 software (StatSoft, Tulsa, OK) for statistical analysis. A Shapiro–Wilk test showed that the distributions of the MMP-9 and IL-8 concentrations were significantly different from normal (*p* < .05); therefore, we used a non-parametric Mann–Whitney *U-*test and Spearman’s rank correlation coefficient (Rs) test in our statistical analysis.

## Results

### IL-8

The IL-8 concentration determined on day 1 was significantly negatively correlated with creatinine clearance early after renal transplantation (on days 1, 7, 14 and 30), as well as during long-term observations (at years 1, 2, 3 and 4) (Table 2). The IL-8 concentration in urine sampled on day 14 after renal transplantation was not significantly correlated with early or long-term creatinine clearance.

**Table 2.** Correlation between GFR and IL-8 in first day after transplantation.

GFR	*N*	Correlation with IL-8 in first day after transplantation
Day 1	61	*p* = .000000026; Rs = −0.64
Day 7	62	*p* = .000021; Rs = −0.51
Day 14	64	*p* = .011; Rs = −0.31
Day 30	58	*p* = .037; Rs = −0.27
Year 1	57	*p* = .13; Rs = −0.20
Year 2	44	*p* = .089; Rs = −0.26
Year 3	36	*p* = .029; Rs = −0.36
Year 4	29	*p* = .019; Rs = −0.43

GFR: glomerular filtration rate.

**Figure 1.**
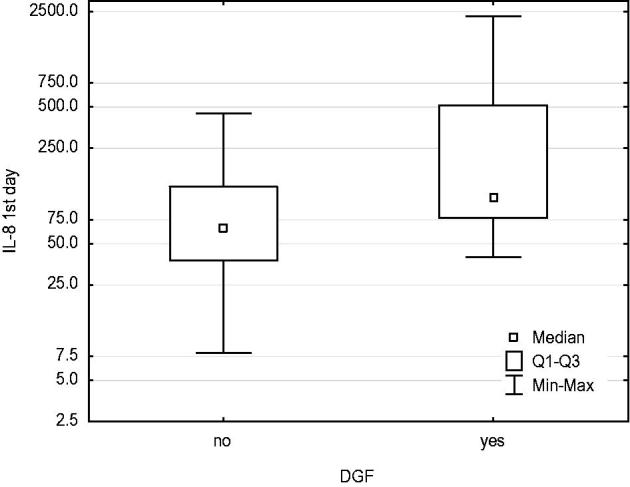
In DGF patients, IL-8 concentration in urine sampled at day 1 was higher than in patients without DGF. Mann–Whitney *U-*test: *p* = .004.

**Figure 2.**
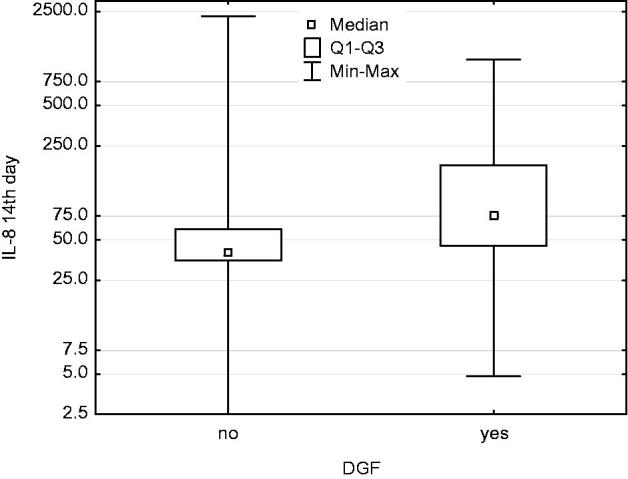
In DGF patients, IL-8 concentration in urine sampled at day 14 was higher than in patients without DGF. Mann–Whitney *U-*test: *p* = .005.

**Figure 3.**
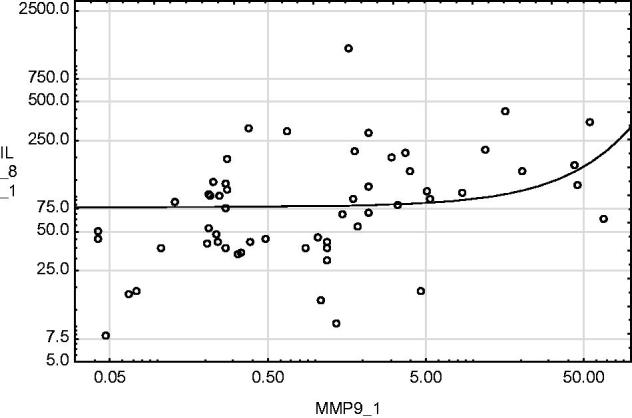
MMP-9 activity determined at day 1 after renal transplantation was positively correlated with IL-8 content determined in urine sampled at day 1. Rs = +0.32, *p* = .015.

**Figure 4.**
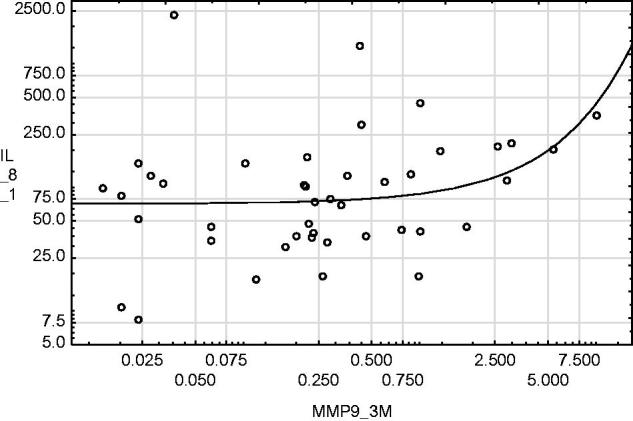
MMP-9 activity determined at month 3 after renal transplantation was positively correlated with IL-8 content determined in urine sampled at day 1. Rs = +0.31, *p* = .036.

IL-8 concentration in urine sampled on day 1 and day 14 was higher in patients demonstrating DGF than in those without DGF (Figures 1 and 2). No relationship was found between IL-8 content and cold ischaemia time. MMP-9 activity determined on day 1 and month 3 after renal transplantation was positively correlated with the IL-8 content determined in urine sampled on day 1, Rs = +0.32, *p* < .05 and Rs = +0.31, *p* < .05, respectively (Figures 3 and 4).

## Discussion

IL-8 is the main chemokine produced by the damaged endothelium and then, after inflammation spreads, by macrophages, T cells and renal tubular epithelial cells [15,16]. IL-8 has a chemotactic effect on neutrophils, inducing them to release lysosomal enzymes and upregulating endothelial adhesion molecules [17,18]. In our study, IL-8 sampled from urine on day 1 was negatively correlated with GFR assessed in the early post-transplant period (days 1, 7, 14 and 30). Furthermore, patients with delayed graft function after transplantation (DGF) had a higher IL-8 concentration in their urine on day 1 and day 14 than the group whose kidneys resumed functioning immediately. This can be explained by the more extensive inflammatory process occurring in the group demonstrating higher IL-8 content, which means larger damage to the renal parenchyma, including renal tubules, and a longer regeneration time. In a rabbit post-reperfusion lung injury model, application of IL-8 antibodies prevented neutrophil infiltration and lung tissue destruction [19]. In their animal model, Mulligan et al. observed that the application of IL-8 antibodies removed the inflammatory infiltration and lung injuries caused by immune complexes. The application of the antibodies prevented neutrophil adhesion and migration to the lung tissue [20]. In research carried out by Kelly et al., in their renal ischemic injury model, the induction of neutropenia led to a lack of typical morphological lesions and good renal function [21]. Similar results were obtained by Hellber and Klausner [22,23]. By inducing neutropenia, Mizutani reduced post-perfusion injury. The same author reduced the level of I-R injury by using the inhibitor elastase, secreted by neutrophils [24]. It can be assumed that IL-8, as the main neutrophil chemokine, is necessary for the inflammatory process to spread from the vessels to the organ parenchyma, exacerbating renal ischaemia. Kwon et al., examined the IL-8 content in urine sampled on day 0 after renal transplantation. They observed that the high IL-8 concentration patients showed characteristics of acute renal failure [25]. Chiao et al. proved that renal damage resulting from I-R was not caused by the ischaemia itself, but rather by the activation of IL-8 and the adhesion molecule ICAM-1. When these two were blocked, renal injury secondary to I-R was completely prevented [26]. IL-8 sampled on day 14 was not correlated with early or long-term creatinine clearance. This could be explained by the nature of the inflammation in the graft changing over time. In inflammatory infiltration, leukocytes other than macrophages and T cells, as well as other cytokines and chemokines, become predominant.

In our study, the IL-8 concentration in urine sampled on day 1 after renal transplantation was negatively correlated with the value for GFR on days 1, 2, 3 and 4 after renal transplantation. IL-8 is known to be a typical chemokine of acute injury, particularly I-R, but not directly responsible for chronic renal injuries, such as parenchymal fibrosis and tubular atrophy. It has been shown that the parenchyma, particularly in the renal tubules, recovers after ischaemia–reperfusion injury. Reparation of tissues damaged as a result of I-R is often improper, which leads to parenchymal fibrosis and tubular atrophy [27,28]. The question is what affects the tubules, determining their reconstructive response or atrophic/fibrotic response [29]. Various mechanisms that lead to such lesions are studied. In rats, after an I-R episode, Gang et al. observed maintained macrophage infiltration that, through the cytokines produced by the macrophages, favoured parenchymal fibrosis and tubular atrophy [30]. Other researchers have shown that maintained oxidative stress is the cause of chronic lesions in the kidney after I-R injury [31]. Furthermore, there is evidence indicating that delayed graft function caused by ischaemia results in worse long-term renal function [32].

In damaged tubular cells, the cell cycle is disordered. It is halted at the G2/M stage, which facilitates the synthesis of TGF-B1 and the connective tissue growth factor (CTGF) [33]. By affecting the existing fibroblasts, these factors activate them, inducing fibrogenesis [34]. Therefore, it can be concluded that at the beginning of the pathway there are IL-8-activated neutrophils and at the end of the pathway there is IF/TA-related renal injury. It appears that the severity of the inflammation that occurs after I-R depends on IL-8 and has an effect on chronic renal function.

Another factor that is undeniably related to I-R damage, particularly to the damage to peritubular capillaries, is metalloproteinases, especially MMP-9 and MMP-2. MMP-9 is a proteolytic enzyme and a member of the gelatinase group. Such enzymes are primarily involved in digesting type IV collagen, as well as lamina, proteoglycans and fibronectin. These proteins are components of the extracellular matrix and the vascular basement membrane. By digesting the basement membrane, they destroy the physiological barrier that makes it impossible for cells to migrate from the vascular lumen to the extravascular space. MMP-9 is indirectly involved in activating neutrophils. It is an activator of IL-8, which is activated by the cleavage of a short amino acid sequence. Moreover, MMP-9 activates a peptide originating from endothelial cells that activates neutrophils. This allows for a further spreading of the inflammatory process [6–8].

Previous studies have shown significant role of IL-8 and MMP-9 in renal transplantation. Elevated IL-8 levels have been observed in human donor allografts with longer ischemic time [35]. Higher levels of urinary IL-8 have been shown in patients who had acute kidney injury after orthotopic liver transplantation [36]. Compared to preimplantation levels, IL-8 transcripts in allograft biopsies were significantly higher one hour after reperfusion in transplanted patients [37]. Borst et al. have shown that reduced levels of IL-8 transcripts in peripheral mononuclear cells predict immediate graft dysfunction and delayed graft function [38]. In the study by Singh, IL-8 –251AA genotype was associated with 2.7-fold increased risk for allograft rejection in recipients experiencing rejection episodes [39]. MMP-9 has been shown to be involved in acute and chronic renal injury along the spectrum of basement membrane damage, to tubular atrophy, to fibrosis, to outright renal failure [40]. MMP-9 concentration in the early post-transplant period is a major marker of early and long-term function of the transplanted kidney. Its increased concentration was correlated with lesions related to tubular atrophy and fibrosis in renal biopsies performed at months 3 and 12 after transplantation. Its concentration is correlated with TGF-β content in a later period. Renal production of MMP-9 on graft reperfusion is associated with cold ischaemia time and emergence of delayed graft function [41]. Turunen et al. have shown that MMP-9 inhibition may offer a means to reduce reperfusion injury in renal transplantation [42]. Mazanowska et al. suggest that proteinuria was significantly associated with increased concentrations of plasma MMP inhibitors and negatively correlated with recipient-estimated glomerular filtration rate (eGFR) [43,44].

Summarizing the above, MMP-9 does not only activate neutrophils, but also facilitates their migration. It has been shown that following reperfusion, a considerable increase in MMP concentration occurs [45]. Inhibiting this increase reduces the scale of renal tissue damage secondary to IRI [46]. Evidence of the cooperation between IL-8 and MMP-9 was found in our results. There is a positive correlation between the IL-8 concentration in urine sampled on day 1 after renal transplantation and the MMP-9 concentration in urine sampled both on day 1 and month 3 after renal transplantation. Research in rheumatoid arthritis has proven that MMP-9 inhibition reduces the production of various inflammatory factors, including IL-8. However, the strength of correlation between IL-8 and MMP-9 concentrations in urine is rather weak and the potential usefulness of their measurement in kidney transplant recipients for clinical practice seems limited.

## Conclusions

A high IL-8 content in urine sampled on day 1 after renal transplantation is an unfavourable marker of early and long-term (years-long) graft function.

A high IL-8 content in urine sampled on day 1 after renal transplantation is positively correlated with the activity of metalloproteinase-9 in urine. This proves that both these chemokines cooperate in the I-R injury of the transplanted kidney.
